# DISSECTING RACIAL DIFFERENCES IN THE PERCEPTION OF DOMAIN-SPECIFIC LIFE SATISFACTION

**DOI:** 10.1093/geroni/igad104.0074

**Published:** 2023-12-21

**Authors:** Timothy Goler, Tirth Bhatta

**Affiliations:** Norfolk State University, Norfolk, Virginia, United States; University of Nevada, Las Vegas, Las Vegas, Nevada, United States

## Abstract

This study examines factors contributing to racial differences in domain-specific life satisfaction. Our study offers a major contribution to prior research that has tended to pay greater attention to psychological well-being outcomes such as life satisfaction which do not directly pertain to material circumstances of older adults. Those studies generally report better life satisfaction and fewer depressive symptoms among Black older adults than their White counterparts. Our study is based on 409 randomly selected older adults from Cleveland (mean age 79 and 25% African American). We considered socioeconomic status, stressors such as recent negative life events and coping resources such as religiosity to understand their impact on domain specific life- satisfaction (e.g., satisfaction with neighborhood, housing, and standard of living). Our ordinary least squares regression estimates suggest that, on average, Black older adults reported lower levels of domain-specific life satisfaction (b=-0.81, p< 0.01) compared to their White counterparts, despite having stronger social support and being more religious. Even though social support and religiosity did not contribute to racial differences in domain specific life satisfaction, the inclusion of education, income, and recent negative life events fully explain such differences. Findings indicate that interrelated factors such as socioeconomic disadvantage and greater life adversities such as negative life events contribute to lower domain life satisfaction among Black older adults. This finding highlights the need for further exploration of factors shaping material-related well-being outcomes for Black older adults. Our study informs future policies addressing racial well-being inequities.

